# Supplementary material for: Monocyte-driven atypical cytokine storm and aberrant neutrophil activation as key mediators of COVID-19 disease severity
Source: Nat Commun. 2021 Jul 5;12:4117. doi: 10.1038/s41467-021-24360-w (PMC8257697; doi:10.1038/s41467-021-24360-w)
Supplement: Supplementary file 1 — Supplementary information [file 41467_2021_24360_MOESM1_ESM.pdf]

Monocyte-driven atypical cytokine storm and aberrant neutrophil activation as key mediators of COVID-19 disease severity

Supplementary Information

Vanderbeke L[1,£], Van Mol P[2,£], Van Herck Y[3,£], De Smet F[4,£], Humblet-Baron S[5,£], Martinod K[6,£] , Antoranz A[4] , Arijs I[2], Boeckx B[2], Bosisio FM[7], Casaer M[8], Dauwe D[8], De Wever W[9], Dooms C[10], Dreesen E[11], Emmaneel A[12], Filtjens J[13], Gouwy M[14], Gunst J[8], Hermans G[8], Jansen S[15], Lagrou K[1], Liston A[16], Lorent N[17], Meersseman P[18], Mercier T[1], Neyts J[15], Odent J[19], Panovska D[4], Penttila PA[20], Pollet E[19] , Proost P[14], Qian J[2], Quintelier K[12], Raes J[21], Rex S[22], Saeys Y[12], Sprooten J[23], Tejpar S[24], Testelmans D[10], Thevissen K[25], Van Buyten T[15], Vandenhaute J[13], Van Gassen S[12], Velásquez Pereira LC[6], Vos R[10], Weynand B[7], Wilmer A[18], Yserbyt J[10], Garg AD[23, #], Matthys P[13,#], Wouters C[5,13,#], Lambrechts D[2,#], Wauters E[10,#,*], Wauters J[18,#]

[1] Laboratory of Clinical Bacteriology and Mycology, Department of Microbiology, Immunology and Transplantation, KU Leuven, Leuven, 3000, Belgium
[2] Laboratory of Translational Genetics, Department of Human Genetics, VIB-KU Leuven, Leuven, 3000, Belgium
[3] Laboratory of Experimental Oncology, Department of Oncology, KU Leuven, Leuven, 3000, Belgium
[4] Laboratory for Precision Cancer Medicine, Translational Cell and Tissue Research, Department of Imaging & Pathology, KU Leuven, Leuven, 3000, Belgium
[5] Adaptive Immunology, Department of Microbiology, Immunology and Transplantation, KU Leuven, Leuven, 3000, Belgium
[6] Centre for Molecular and Vascular Biology, Department of Cardiovascular Sciences, KU Leuven, Leuven, 3000, Belgium
[7] Translational Cell & Tissue Research, Department of Imaging & Pathology, KU Leuven, Leuven, 3000, Belgium
[8] Laboratory of Intensive Care Medicine, Department of Cellular and Molecular Medicine, KU Leuven, Leuven, 3000, Belgium
[9] Radiology, Department of Imaging & Pathology, KU Leuven, Leuven, 3000, Belgium
[10] Laboratory of Respiratory Diseases and Thoracic Surgery (BREATHE), Department of Chronic Diseases and Metabolism, KU Leuven, Leuven, 3000, Belgium
[11] Clinical Pharmacology and Pharmacotherapy, Department of Pharmaceutical and Pharmacological Sciences, KU Leuven, Leuven, 3000, Belgium
[12] Department of Applied Mathematics, Computer Science and Statistics, VIB-UGent Center for Inflammation Research, VIB-UGent, Gent, 9000, Belgium
[13] Laboratory of Immunobiology, Department of Microbiology, Immunology and Transplantation, Rega Institute, KU Leuven, Leuven, 3000, Belgium
[14] Laboratory of Molecular Immunology, Department of Microbiology, Immunology and Transplantation, Rega Institute, KU Leuven, Leuven, 3000, Belgium
[15] Laboratory of Virology and Chemotherapy, Department of Microbiology, Immunology and Transplantation, Rega Institute, KU Leuven, B Leuven, 3000, Belgium
[16] Laboratory of Lymphocyte Signalling and Development, The Babraham Institute, Babraham Research Campus, Cambridge, CB22 3AT, United Kingdom
[17] Department of Pneumology, University Hospitals Leuven, Leuven, 3000, Belgium
[18] Laboratory for Clinical Infectious and Inflammatory Disorders, Department of Microbiology, Immunology and Transplantation, KU Leuven, Leuven, 3000, Belgium
[19] Department of Internal Medicine, University Hospitals Leuven, Leuven, 3000, Belgium
[20] KU Leuven Flow & Mass Cytometry Facility, KU Leuven, Leuven, 3000, Belgium
[21] Laboratory of Molecular Bacteriology (Rega Institute), Department of Microbiology, Immunology and Transplantation, KU Leuven, and VIB Center for
Microbiology, Leuven, 3000, Belgium
[22] Anesthesiology and Algology, Department of Cardiovascular Sciences, KU Leuven, Leuven, 3000, Belgium
[23] Laboratory for Cell Stress & Immunity (CSI), Department of Cellular and Molecular Medicine (CMM), KU Leuven, Leuven, 3000, Belgium
[24] Molecular Digestive Oncology, Department of Oncology, KU Leuven, Leuven, 3000, Belgium
[25] Centre of Microbial and Plant Genetics, Department of Microbial and Molecular Systems (M2S), KU Leuven, Leuven, 3000, Belgium

[£] equal contribution

[#] joint supervision

[*]corresponding author/Lead Contact: els.wauters@kuleuven.be

Supplementary Table 1: **Laboratory findings of patients infected with MAS versus COVID-19 critical condition and within COVID-19 clinical condition subgroups.**

	**MAS (n=10)**	**COVID-19 Critical clinical condition (n=22)**	**p-value MAS-COVID-19 critical**	**COVID-19 Mild-Moderate clinical condition (n=39)**	**p-value COVID-19 Critical-Mild-Moderate**
C-reactive protein, mg/L	91,7 [42,4-272,5]	159,5 [75,4-252,4]	0,268	68,1 [27,6-127,1]	**0,0005**
Ferritin, µg/L	18529 [4238-34858]	1406 [682-3974]	**0,0004**	843 [322-1543]	**0,036**
White blood cell count, $\times 10^9$/L	2,94 [0,99-14,33]	8,57 [6,75-10,13]	0,077	5,73 [4,03-8,93]	**0,012**
Neutrophil count, $\times 10^9$/L	2,7 [0,7-10,9]	6,1 [5,3-8,9]	0,081	4,3 [3,2-7,0]	**0,023**
Total lymphocyte count, $\times 10^9$/L	0,40 [0,20-1,65]	0,9 [0,7-1,3]	0,216	1,1 [0,8-1,7]	0,214
Neutrophil-to-lymphocyte ratio	4,60 [2,53-8,12]	8,07 [4,59-10,85]	0,128	3,33 [2,44-5,95]	**0,002**
Platelet count, $\times 10^9$/L	89 [31-215]	247 [191-355]	**0,010**	239 [177-288]	0,383
AST, U/L	156 [46-353]	59 [37-98]	0,132	37 [24-61]	**0,024**
ALT, U/L	67 [16-104]	47 [28-77]	0,726	33 [20-64]	0,167
D-dimers, µg/L	7602 [2330-7650]	1144 [735-2125]	**0,0007**	832 [541-1292]	0,058
Fibrinogen, g/L	3,27 [1,80-7,31]	5,62 [5,25-6,61]	0,079	5,30 [4,29-6,51]	0,274
Lactate dehydrogenase, U/L	868 [505-1546]	416 [352-567]	**0,006**	298 [211-396]	**0,0002**
Creatine kinase, U/L	40 [20-754]	118 [72-264]	0,277	78 [50-181]	0,116
Triglycerides, mmol/L	3,48 [1,73-4,44]	1,58 [1,14-2,17]	**0,034**	1,31 [1,05-1,76]	0,228

Data are median [IQR]. The p-values comparing COVID-19 critical condition and MAS, and the p-values comparing mild-moderate and critical clinical condition are from a two-sided Mann-Whitney U test. ALT: alanine aminotransferase; AST: aspartate aminotransferase; MAS: macrophage activation syndrome. Bold font is used to highlight statistically significant findings.

Supplementary Table 2: **Demographics of MAS and non-COVID pneumonia control groups.**

	**MAS patients (n=10)**	**NON-COVID pneumonia (n=11)**
Age, years	22 [8-53]	67 [55-73]
Sex	..	..
Men	4	6
Women	6	5
Comorbidity	..	..
Arterial hypertension	2 (20)	7 (64)
Diabetes mellitus	0 (0)	3 (27)
Chronic kidney failure	0 (0)	3 (27)
Atrial fibrillation	0 (0)	1 (9)
Obesity	2 (20)	1 (9)
Haematological malignancy	1 (10)	1 (9)
Oncological malignancy	1 (10)	7 (64)
Respiratory support	3 (30)	7 (64)
Oxygen via nasal cannula	2 (20)	6 (55)
High flow oxygen support	0 (0)	0 (0)
Invasive ventilation	0 (0)	1 (9)
Prone ventilation	1 (10)	0 (0)

Data are median [IQR], or n (%). Chronic kidney failure is defined as eGFR <60mL/min/1.73m2 during 3 months or structural renal disease under nephrology follow-up. Obesity is defined according to WHO definitions (BMI $\geq$ 30 kg/m^2 if >19 years old, BMI-for-age > 2 SD above WHO growth reference median for patients aged 5-19 years old and BMI-for-age > 3 SD above WHO child growth standards median for patients <5 years old). BMI: body mass index; MAS: macrophage activation syndrome; SD: standard deviation; WHO: world health organization.

Supplementary Table 3: **Assessment of impact of demographic variables on key cytokine levels, comparing MAS and critical COVID-19, using linear regression.**

**CXCL8**						
Source	Value	Standard error	t	Pr > |t|	Lower bound (95%)	Upper bound (95%)
AGE	-0,129	0,230	-0,562	0,579	-0,600	0,342
GROUP-Critical COVID-19	0,614	0,230	2,674	**0,013**	0,143	1,085
GROUP-MAS	0,000	0,000				
Source	Value	Standard error	t	Pr > |t|	Lower bound (95%)	Upper bound (95%)
GROUP-Critical COVID-19	0,468	0,165	2,843	**0,008**	0,130	0,806
GROUP-MAS	0,000	0,000				
SEX-F	-0,213	0,165	-1,291	0,208	-0,551	0,125
SEX-M	0,000	0,000				
Source	Value	Standard error	t	Pr > |t|	Lower bound (95%)	Upper bound (95%)
GROUP-Critical COVID-19	0,525	0,168	3,125	**0,004**	0,180	0,870
GROUP-MAS	0,000	0,000				
CKD-0	0,011	0,168	0,066	0,948	-0,334	0,356
CKD-1	0,000	0,000				
**IFN-ɣ**						
Source	Value	Standard error	t	Pr > |t|	Lower bound (95%)	Upper bound (95%)
AGE	0,002	0,217	0,010	0,992	-0,441	0,445
GROUP-Critical COVID-19	-0,577	0,217	-2,666	**0,012**	-1,020	-0,134
GROUP-MAS	0,000	0,000				
Source	Value	Standard error	t	Pr > |t|	Lower bound (95%)	Upper bound (95%)
GROUP-Critical COVID-19	-0,618	0,155	-3,987	**0,000**	-0,936	-0,300
GROUP-MAS	0,000	0,000				
SEX-F	-0,353	0,155	-2,281	**0,031**	-0,671	-0,035
SEX-M	0,000	0,000				
Source	Value	Standard error	t	Pr > |t|	Lower bound (95%)	Upper bound (95%)
GROUP-Critical COVID-19	-0,552	0,166	-3,323	**0,003**	-0,893	-0,211
GROUP-MAS	0,000	0,000				
CKD-0	-0,114	0,166	-0,687	0,498	-0,455	0,227
CKD-1	0,000	0,000				
**VEGF**						
Source	Value	Standard error	t	Pr > |t|	Lower bound (95%)	Upper bound (95%)
AGE	-0,082	0,229	-0,359	0,722	-0,551	0,387
GROUP-Critical COVID-19	0,555	0,229	2,421	**0,022**	0,086	1,024
GROUP-MAS	0,000	0,000				
Source	Value	Standard error	t	Pr > |t|	Lower bound (95%)	Upper bound (95%)
GROUP-Critical COVID-19	0,441	0,172	2,565	**0,016**	0,088	0,794
GROUP-MAS	0,000	0,000				
SEX-F	-0,154	0,172	-0,896	0,378	-0,507	0,199
SEX-M	0,000	0,000				
Source	Value	Standard error	t	Pr > |t|	Lower bound (95%)	Upper bound (95%)
GROUP-Critical COVID-19	0,488	0,173	2,822	**0,009**	0,133	0,842
GROUP-MAS	0,000	0,000				
CKD-0	0,031	0,173	0,177	0,861	-0,324	0,385
CKD-1	0,000	0,000				

Chronic kidney disease is defined as eGFR <60mL/min/1.73m2 during 3 months or structural renal disease under nephrology follow-up. CKD: Chronic kidney disease.

Supplementary figure 1: **Cytokine and chemokine plasma levels: comparison with MAS.**

Overview of cytokine and chemokine levels with comparison between healthy controls (HC, n=10), COVID-19 critical condition (CCC, n=22) and MAS (n=10) patients. Plasma concentrations were measured by MSD (Meso Scale Discovery). Boxplot representation (center line, mean; box limits, upper and lower quartiles; whiskers, range; points, data points per patient). A two-sided Kruskal-Wallis test with Dunn's correction for multiple comparisons was used; **IL-1a**: p=0.003 HC vs MAS; **TNF-b**: p=0.024 CCC vs MAS; **CCL3**: p=0.0008 HC vs CCC, p<0.0001 HC vs MAS; **CCL4**: p=0.009 HC vs CCC, p=0.0002 HC vs MAS; **CCL13**: p=0.021 CCC vs MAS; **CCL17**: p=0.044 HC vs MAS; **CCL26**: p=0.006 HC vs CCC, p=0.019 HC vs MAS; **CCL2**: p<0.0001 HC vs CCC, p=0.0001 HC vs MAS; **CXCL10**: p<0.0001 HC vs CCC, p<0.0001 HC vs MAS; **IL-5**: p=0.0005 HC vs CCC, p=0.035 CCC vs MAS; **IL-7**: p=0.005 HC vs CCC, p=0.0009 CCC vs MAS; **IL-10**: p=0.0003 HC vs CCC, p<0.0001 HC vs MAS; **IL-13**: p=0.040 HC vs MAS; **IL-15**: p<0.0001 HC vs CCC, p=0.0004 HC vs MAS; **IL-17A**: p=0.020 HC vs CCC. Significance is shown as * p < 0.05 ; ** p < 0.01 ; *** p < 0.001 and **** p < 0.0001. MAS= macrophage activation syndrome. Source data are provided as a Source Data file.

Supplementary figure 2: **Cytokine and chemokine plasma levels: comparison within COVID-19 subgroups.**

Overview of cytokine and chemokine levels with comparison between COVID-19 patients in mild-moderate (CMM, n=39) and critical (CCC, n=22) clinical condition versus healthy controls (HC, n=10). Plasma concentrations were measured by MSD (Meso Scale Discovery). Boxplot representation (center line, mean; box limits, upper and lower quartiles; whiskers, range; points, data points per patient). A two-sided Kruskal-Wallis test with Dunn's correction for multiple comparisons was used; **CCL4**: p=0.001 HC vs CCC, p=0.004 CMM vs CCC; **CCL22**: p=0.041 HC vs CCC; **CCL26**: p=0.029 HC vs CMM, p=0.007 HC vs CCC; **CXCL9**: p=0.028 HC vs CMM, p=0.003 HC vs CCC; **CXCL10**: p=0.0005 HC vs CMM, p<0.0001 HC vs CCC; **IL-5**: p=0.030 HC vs CMM, p=0.0001 HC vs CCC; p=0.042 CMM vs CCC; **IL-7**: p=0.001 HC vs CCC, p=0.008 CMM vs CCC; **IL-10**: p=0.001 HC vs CMM, p<0.0001 HC vs CCC; **IL-12/IL-23p40**: p=0.0498 HC vs CMM; **IL-13**: p=0.0007 HC vs CMM, p=0.039 CMM vs CC; **IL-15**: p=0.0004 HC vs CMM, p<0.0001 HC vs CCC, p=0.025 CMM vs CCC; **IL-17A**: p=0.006 HC vs CMM, p= 0.009 HC vs CCC. Significance is shown as * p < 0.05 ; ** p < 0.01 ; *** p < 0.001, **** p < 0.0001. Source data are provided as a Source Data file.

Supplementary figure 3: **Computational and systems biology prediction analysis of COVID-19 hypercytokinemia and immune cell phenotypes.**

(a) Gene correlation network analyses for indicated cytokines/chemokines-coding genes per (indicated) immune cell-type, comparing mild-moderate and critical COVID-19 patient subgroups. Connected edges represent tendency of particular genes to be co-expressed/correlated in terms of parallel expression (based on the extensive reference immune cell gene-expression profiles) and the higher thickness of the edges indicates higher statistical significance. (b) Gene Ontology (GO)net-driven Euler network analyses of cytokines-coding genes (correlating with particular immune cell types clusters) integrating GO Biological Processes terms. Yellow GO terms indicate those connected with (from top to bottom) all 3, all 2, at least 3 or at least 5 cytokines or chemokines. Blue GO terms indicate those connected with less target than these. Herein, our input human genes were computed for GO biological process term annotation based on predefined GO slim subset for immunology (experimental; process only) and represented via the Euler force-directed (physics simulation) layout (wherein gene-unconnected terms were hidden). This analysis reconstructs relationship between genes and GO terms thereby giving a better idea of the functional immunological impact of specific input genes.

Supplementary figure 4: **ScRNAseq data of COVID-19 PBMCs.**

(a) UMAP plot of 83,524 single-cells (from 23 patients, n=13 for mild-moderate, n=10 for critical), colour-coded per cell type (top), per patient (middle) and per clinical status (bottom). (b) Expression of marker genes projected on UMAP. Composition of the marker genes: B-cell (*CD79A*), plasma cell (*JCHAIN*), erythrocyte (*HBB*), thrombocyte (*PPBP*), DC (*LILRA4, FCER1A*), myeloid cell (*CD68*), T-cell (*CD3E*), CD8+ T-cell (*CD3E, CD8*), CD4+ T-cell (*CD3E, CD4*) NK-cell (*FCGR3A*), proliferative cell (*STMN1*). (c) Heatmap of marker genes per immune cell type. (d) Relative immune cell type abundance in peripheral blood of COVID-19 patients, comparing mild-moderate to critical cases. Boxplot representation (center line, mean; box limits, upper and lower quartiles; whiskers, range; points, data points per patient). A two-sided Wilcoxon rank sum test was used; CD4+ T-cells p=0.0422, CD8+ T-cells p=0.0213, non-classical monocytes p=0.0009, plasmacells p=0.0003, thrombocytes p=0.0025. (e) Violin plots of expression level of cytokine/chemokine/chemokine receptor/transcription factor coding genes in PBMCs of COVID-19 patients. (f) Feature plots of expression level of cytokine/chemokine/chemokine receptor/transcription factor coding genes in PBMCs of COVID-19 patients. Significance is shown as * $p < 0.05$; ** $p < 0.01$; *** $p < 0.001$. Source data are provided as a Source Data file.

Supplementary Figure 5: **Mass cytometry clustering analysis overview of the non-granulocyte population.**

(a) Heat map representation of the median expression values of each marker across the 107 defined FlowSOM clusters. The color code in the first row highlights the various cell types, while the clusters annotations are shown on the right. (b-c) Spade tree representation of the same clustering analysis, color-coded by the identified cell populations (b), with the relative expression levels of all 33 included markers across the clusters (c). The numbers in the Spade tree correspond to the clusters referred to in the manuscript. Source data are provided as a Source Data file.

Supplementary Figure 6: **Flow cytometry gating of COVID-19 PBMC.**

(a) Comparison of T-helper 1 subset (defined as CD4+ T-cells secreting IFN-γ) in healthy controls (n=6) and COVID-19 patients (n=43) based on flow cytometric analyses of PBMCs. Boxplot representation (center line, mean; box limits, upper and lower quartiles; whiskers, range; points, data points per patient). A two-sided Wilcoxon rank sum test was used; p=0.040. Source data are provided as a Source Data file. (b) PD-1 expression on CD8+ TEMRA cells in healthy (HC, n=6), mild-moderate (CMM, n=23) and critical (CCC, n=20) groups based on flow cytometric analyses of PBMCs. Boxplot representation (center line, mean; box limits, upper and lower quartiles; whiskers, range; points, data points per patient). A two-sided Wilcoxon rank sum test with Benjamini-Hochberg correction for multiple group comparisons was used; p=0.024 HC vs CCC, p=0.007 CMM vs CCC. Source data are provided as a Source Data file. Significance is shown as * $p < 0.05$; ** $p < 0.01$. (c) Manual gating strategy of flow cytometric PBMC data: representative plots for the identification of HLA-DR on monocytes. (d) Representative plots for the identification of interferon gamma secreting CD4 T cells. (e) Representative plots for the identification of PD1+ TEMRA CD8 T cells.

Supplementary figure 7: **Neutrophil activation and NET analysis in peripheral blood.** Comparison between healthy (HC, n=9) and COVID-19 (COV, n=52) patients of MPO-levels (a), MPO-DNA complexes (b) and citrullinated histone 3 (c). Same analyses performed in mild-moderate COVID-19 patients (CMM, n=34) compared to non-COVID pneumonia patients with comparable clinical characteristics and levels of respiratory support (nonCOV, n=6) (d,e,f) and within COVID-19 subgroups (n=34 for mild-moderate (CMM), n=18 for critical (CCC)) (g,h,i). Correlation analysis of MPO-DNA and neutrophil count of COVID-19 cases (j) as well as MPO-DNA and MPO (k). H3Cit = citrullinated histone 3; MPO: myeloperoxidase; NPP = normalised to plasma pool. Boxplot representation (center line, mean; box limits, upper and lower quartiles; whiskers, range; points, data points per patient). Mann Whitney T test (a-i) and simple linear regression analysis (j, k) were used, all statistical significance testing was based on a two-sided hypothesis. Source data are provided as a Source Data file. **HC vs COV**: $p<0.0001$ MPO (a), $p=0.0005$ MPO-DNA (b), $p=0.0001$ H3Cit (c); **CMM vs nonCOV**: $p=0.049$ MPO (d), $p=0.021$ MPO-DNA (e), $p=0.008$ H3Cit (f); **CM vs CCC**: $p=0.013$ MPO (g), $p=0.016$ MPO-DNA (h), $p=0.103$ H3Cit (i). Significance is shown as * $p < 0.05$; ** $p < 0.01$; *** $p < 0.001$; **** $p < 0.0001$.

Supplementary figure 8: **Contribution of neutrophils to lung inflammation in COVID-19.**

(a) UMAP plot of 26,605 single-cells (from 11 patients, n=6 for COVID-19, n=5 for non-COVID pneumonia), colour-coded per cell type (top), per patient (middle) and per underlying pathology (bottom). (b) Expression of marker genes projected on UMAP. Composition of the marker genes: secretory epithelial cell (*SCGB1A1*), basal cell (*KRT5*), KRT4+/KRT13+ epithelial cell (*KRT13*), ciliated epithelial cell (*TPPP3*), FCN1+ monocyte (*FCN1*), RGS1+ macrophage (*RGS1*), CXCL10+ macrophage (*CXCL10*), alveolar macrophage (*FABP4*), neutrophil (*FCGR3B*), CD4+ T-cell (*CD3D, CD4*), CD8+ T-cell (*CD3D, CD8*), NK-cell (*NCAM1*), B-cell (*CD79A*). (c) Heatmap of marker genes per immune cell type. (d) Violin plots of expression level of cytokine/chemokine/chemokine receptor/transcription factor coding genes in BAL fluid cell populations. (e) Feature plots of expression level of cytokine/chemokine/chemokine receptor/transcription factor coding genes in BAL fluid cell populations. (f) Heatmap of differentially expressed genes, comparing the active and resting neutrophil subpopulations. 'Red' indicates relative upregulation, 'blue' indicates relative downregulation. (g) NET forming activity in active versus resting neutrophils, based on relative upregulation of NET formation associated genes. A two-sided Wilcoxon rank sum test was used. Significance is shown as **** $p < 0.0001$. Source data are provided as a Source Data file.

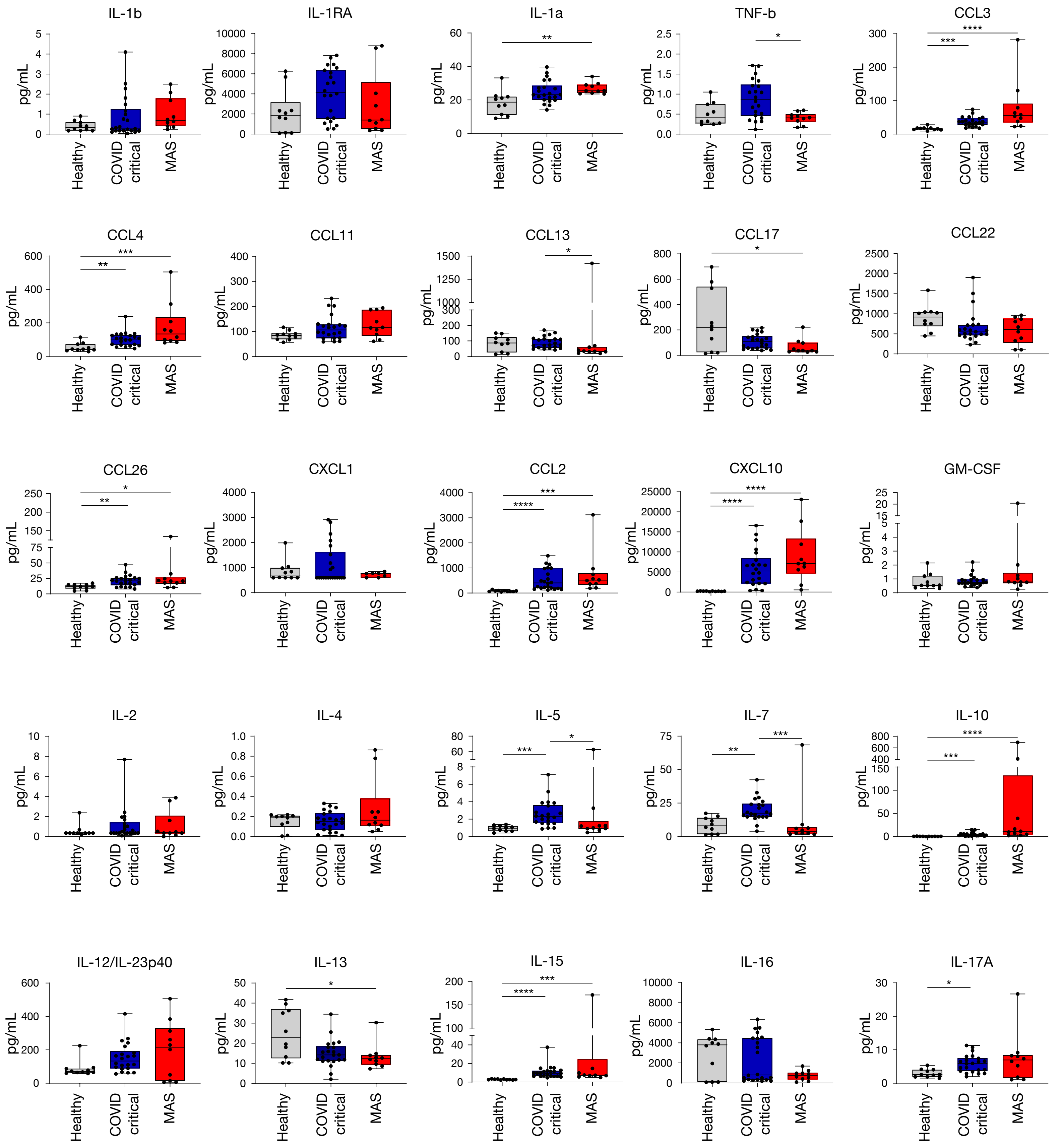

Supp. Figure 1

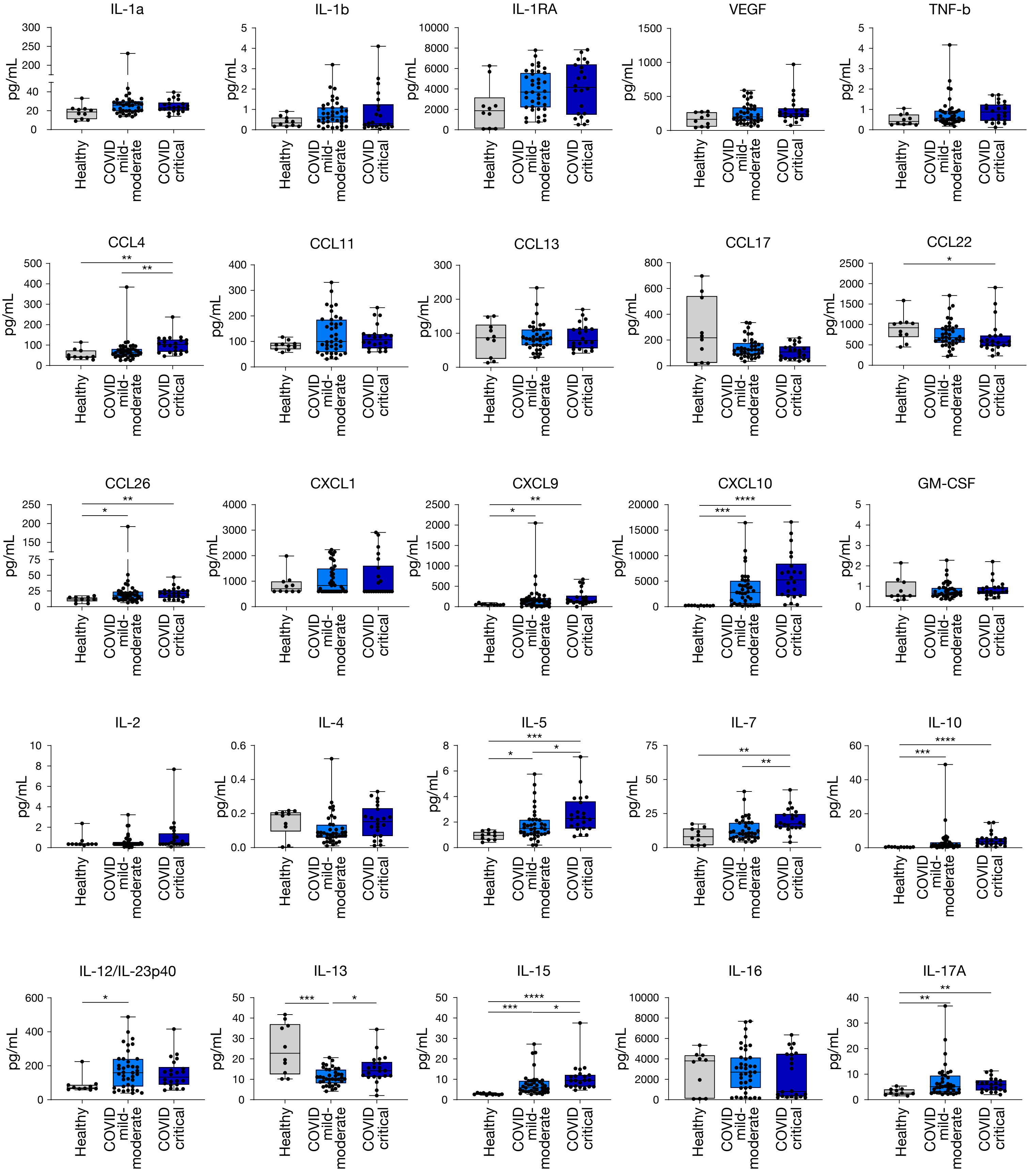

Supp. Figure 2

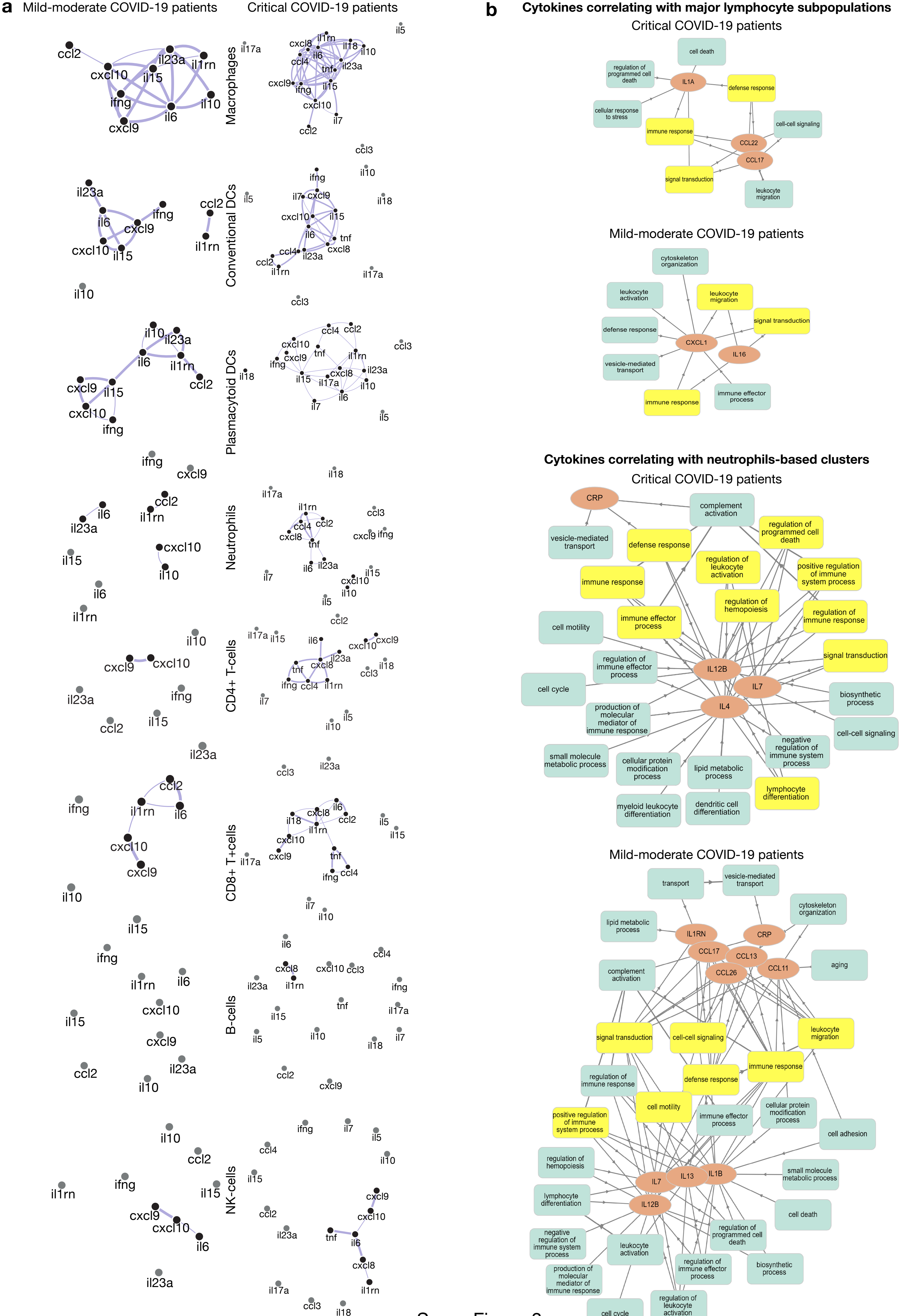

Supp. Figure 3

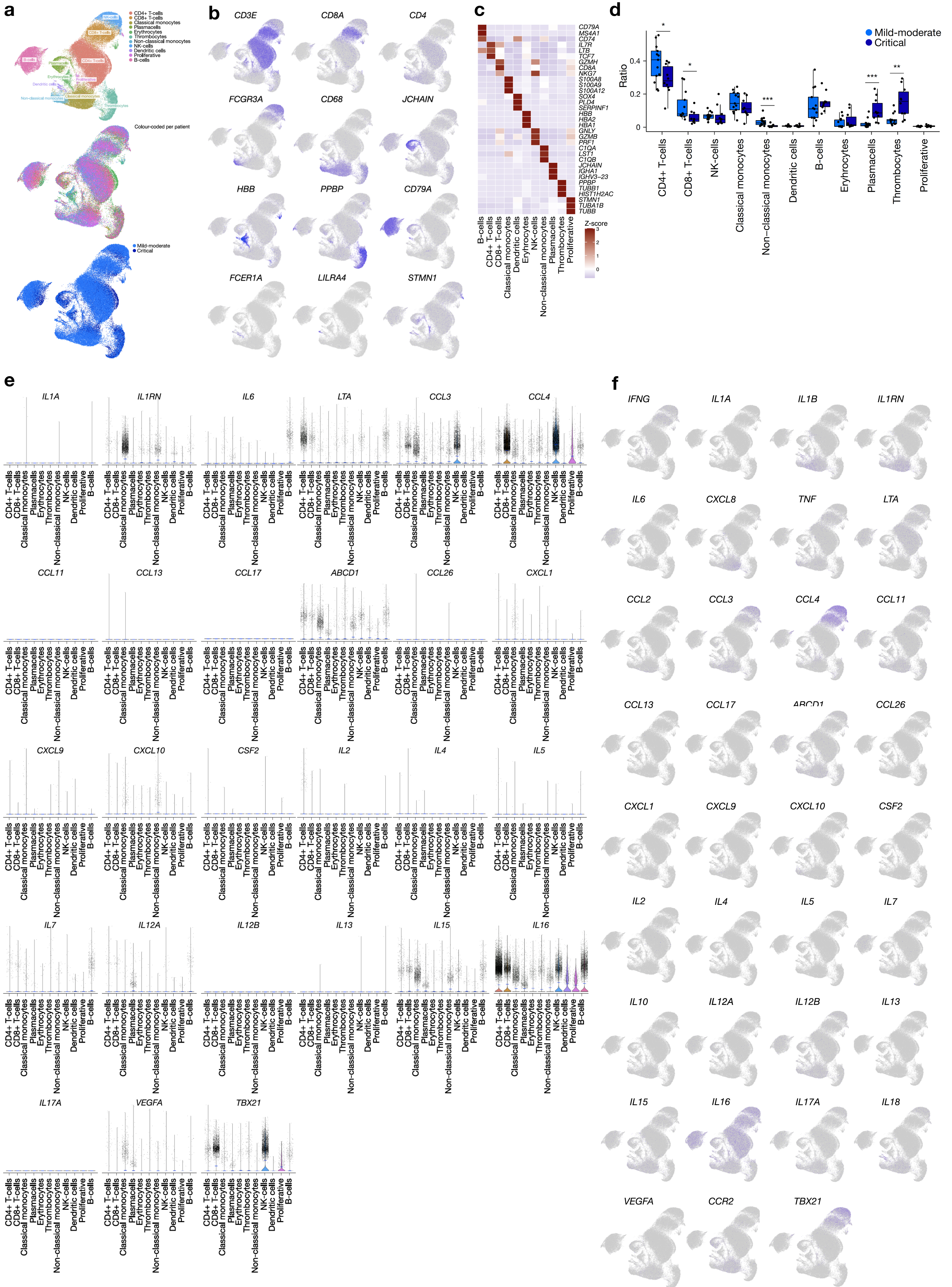

Supp. Figure 4

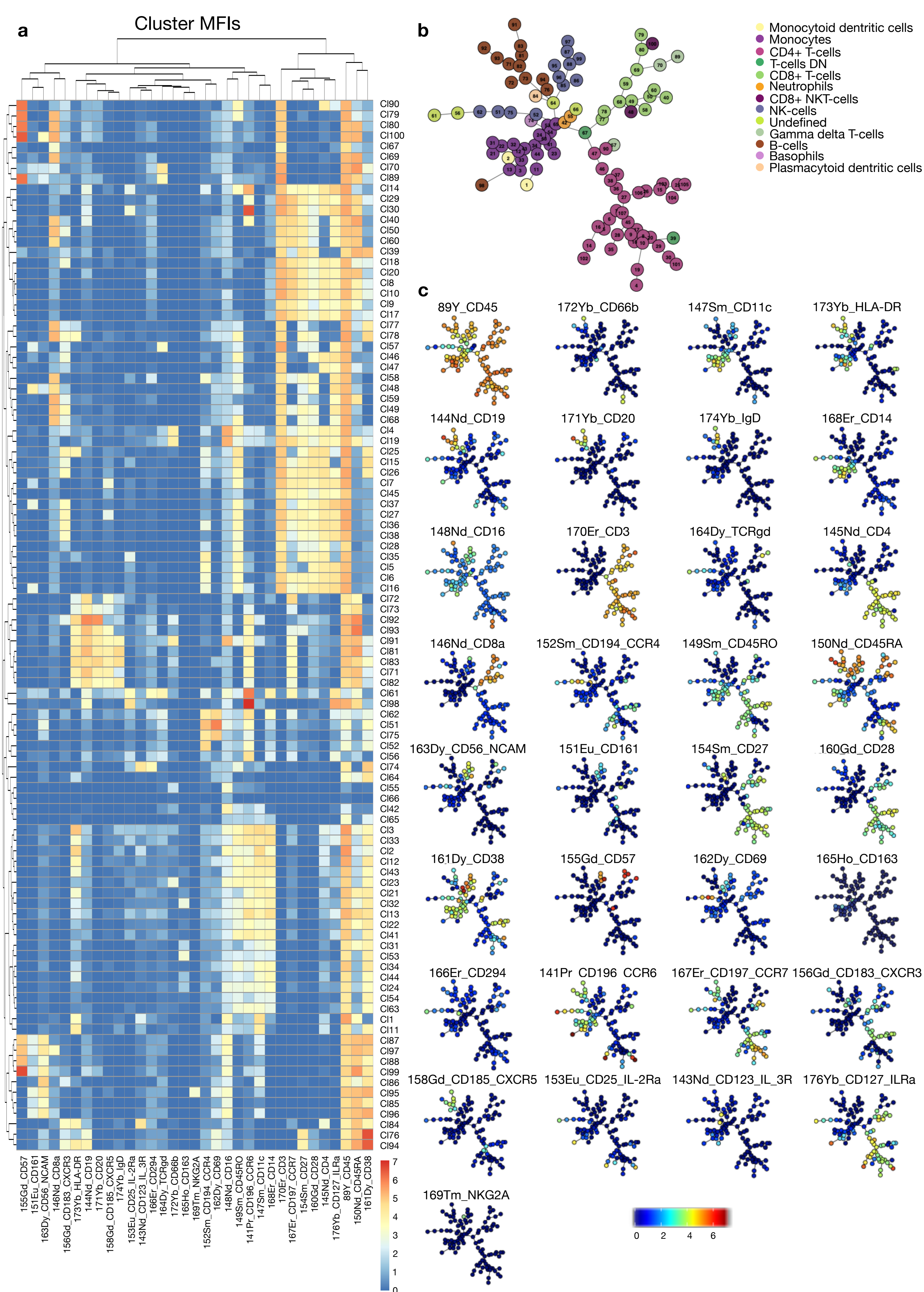

Supp. Figure 5

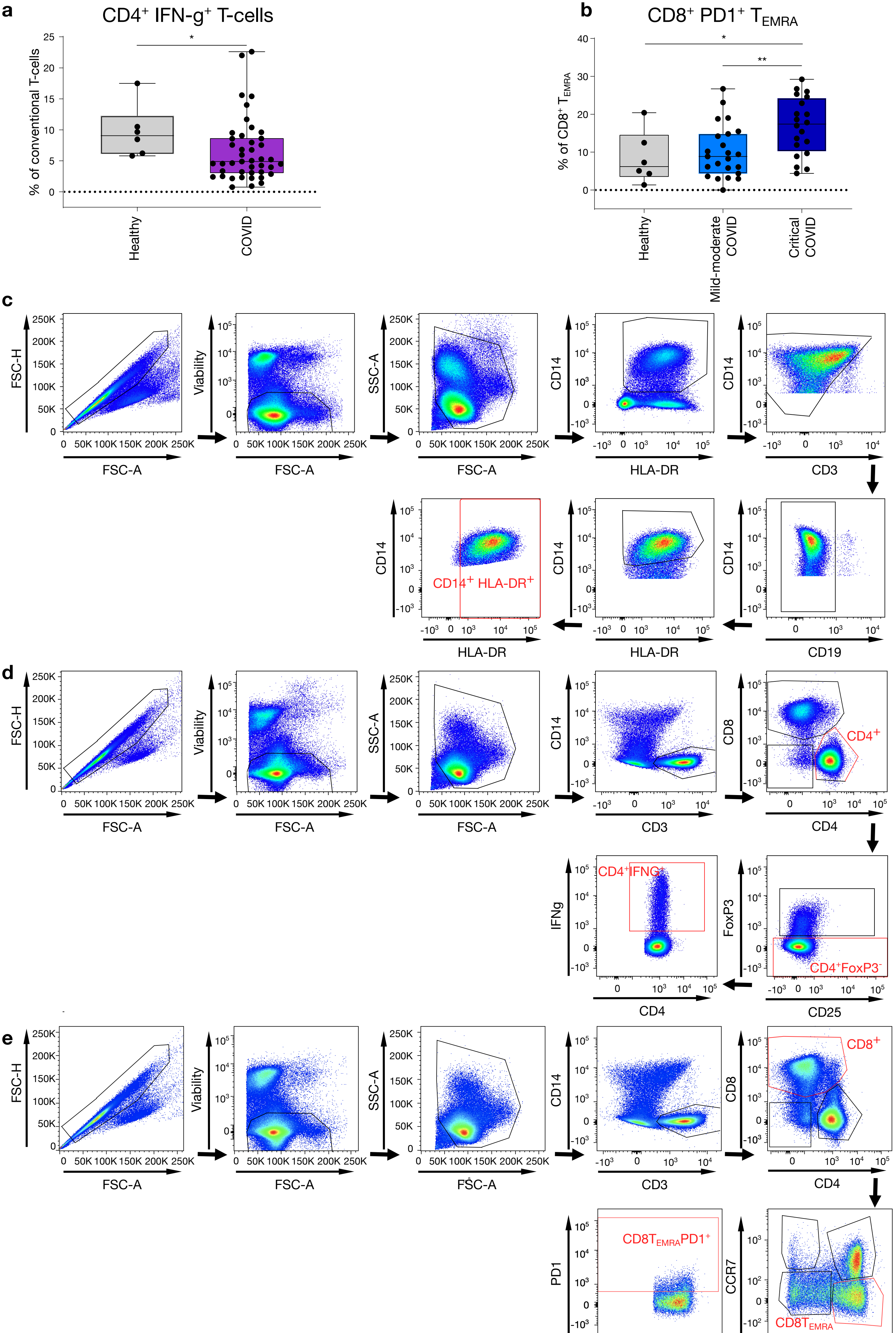

Supp. Figure 6

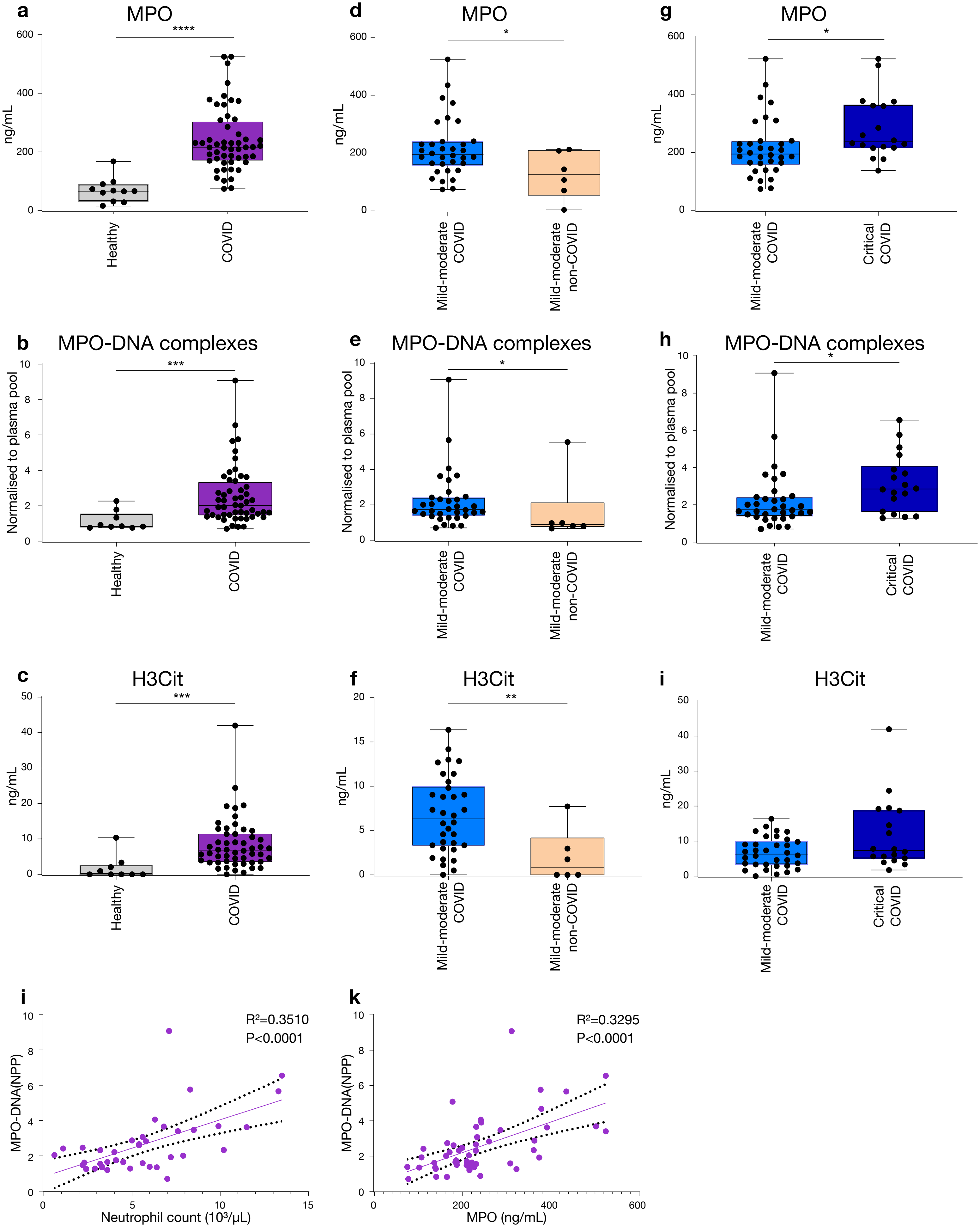

Supp. Figure 7

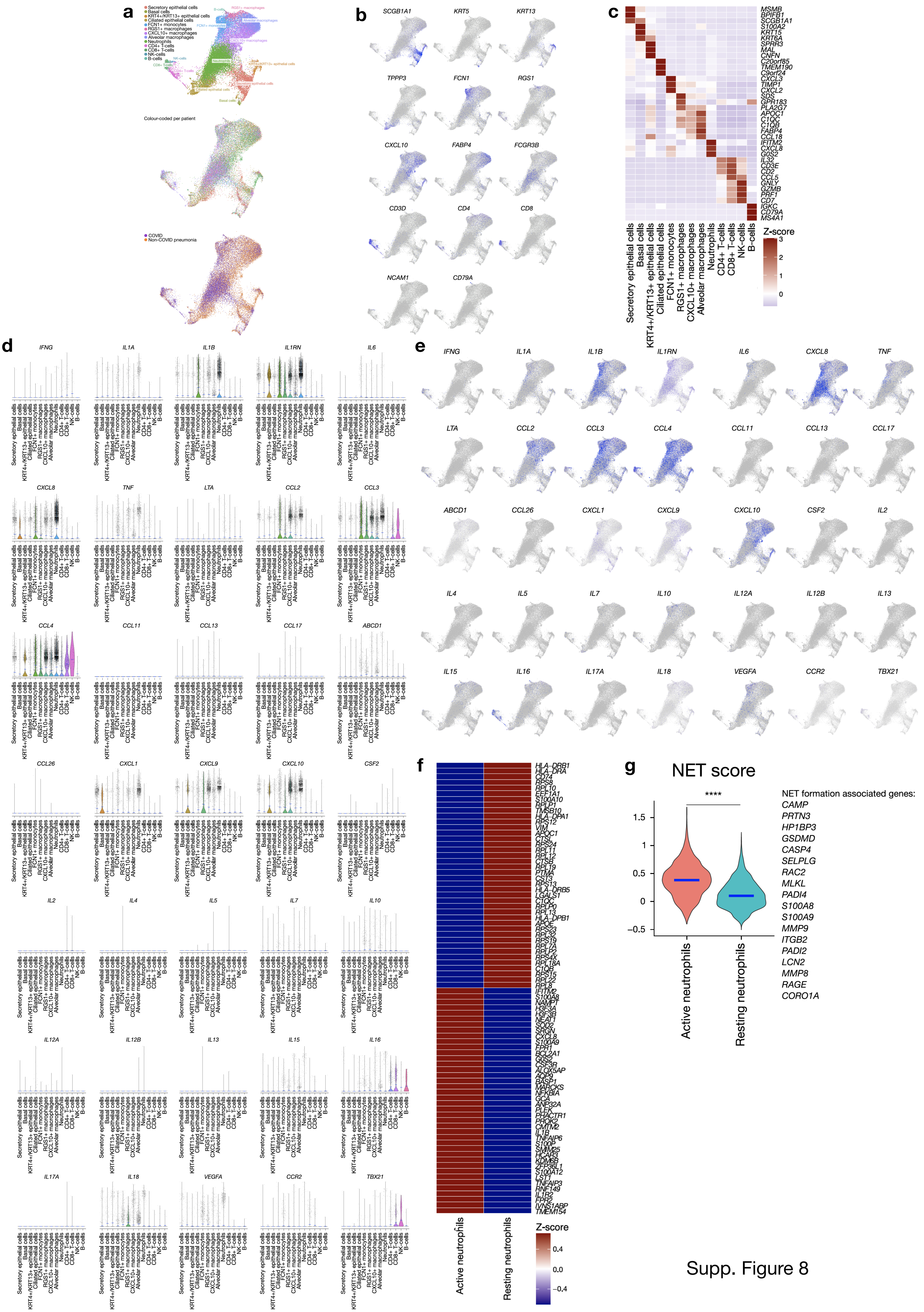

Supp. Figure 8
