# Supplementary material for: Monocyte-driven atypical cytokine storm and aberrant neutrophil activation as key mediators of COVID-19 disease severity
Source: Nat Commun. 2021 Jul 5;12:4117. doi: 10.1038/s41467-021-24360-w (PMC8257697; doi:10.1038/s41467-021-24360-w)
Supplement: Supplementary file 3 — Reporting summary [file 41467_2021_24360_MOESM3_ESM.pdf]

nature research

Corresponding author(s): Els Wauters

Last updated by author(s): May 7, 2021

Reporting Summary

Nature Research wishes to improve the reproducibility of the work that we publish. This form provides structure for consistency and transparency in reporting. For further information on Nature Research policies, see our Editorial Policies and the Editorial Policy Checklist.

Statistics

For all statistical analyses, confirm that the following items are present in the figure legend, table legend, main text, or Methods section.

n/a	Confirmed	
☐	☒	The exact sample size (*n*) for each experimental group/condition, given as a discrete number and unit of measurement
☐	☒	A statement on whether measurements were taken from distinct samples or whether the same sample was measured repeatedly
☐	☒	The statistical test(s) used AND whether they are one- or two-sided<br>*Only common tests should be described solely by name; describe more complex techniques in the Methods section.*
☐	☒	A description of all covariates tested
☐	☒	A description of any assumptions or corrections, such as tests of normality and adjustment for multiple comparisons
☐	☒	A full description of the statistical parameters including central tendency (e.g. means) or other basic estimates (e.g. regression coefficient) AND variation (e.g. standard deviation) or associated estimates of uncertainty (e.g. confidence intervals)
☐	☒	For null hypothesis testing, the test statistic (e.g. *F*, *t*, *r*) with confidence intervals, effect sizes, degrees of freedom and *P* value noted<br>*Give P values as exact values whenever suitable.*
☒	☐	For Bayesian analysis, information on the choice of priors and Markov chain Monte Carlo settings
☒	☐	For hierarchical and complex designs, identification of the appropriate level for tests and full reporting of outcomes
☐	☒	Estimates of effect sizes (e.g. Cohen's *d*, Pearson's *r*), indicating how they were calculated

Our web collection on statistics for biologists contains articles on many of the points above.

Software and code

Policy information about availability of computer code

Data collection	- Demographic, clinical, laboratory, radiologic, treatment and outcome data from patient electronic medical records were obtained through a standardized research form in Research Electronic Data Capture Software (REDCAP v.10.6.13, Vanderbilt University).<br>- FACS data were acquired on a BD Symphony.<br>- CyTOF software version 6.7.1016 and the Maxpar Direct Immune Profiling Assay.tem template were used to acquire and normalize data from the stained CyTOF samples.<br>- scRNA-seq libraries were sequenced on an Illumina NovaSeq 6000, and mapped to the human genome GRCh38 using CellRanger (10x Genomics).

Data analysis	- Statistical analyses were performed using R (version 3.6.3 and 4.0, R Foundation for Statistical Computing, R Core Team, Vienna, Austria) in the RStudio integrated development environment (version 2.2.1; RStudio, Inc., Boston, MA, USA) and Graphpad Prism version 8.4.2.<br>- To create similarity matrix analyses between cytokine/chemokine data and mass cytometry-derived peripheral immune cell enrichments per COVID-19 patient subgroup (i.e. mild-moderate vs. severe), we utilized the Morpheus software (version 1, https://software.broadinstitute.org/morpheus and https://github.com/cmap/morpheus.js).<br>- For network analyses integrating the Gene Ontology (GO) terms specific for immunology-related biological processes, we entered the specified genes into the GOnet computational pipeline (Ontology version: 2019-07-01 and Human annotation version: 2019-07-01).<br>- For flowcytometry analyses, manual gating has been performed with FlowJo (version 10.6.1). Compensation was performed using Autospill ref: doi: https://doi.org/10.1101/2020.06.29.177196.<br>- Normalized CyTOF .fcs files were transferred to the Maxpar Pathsetter™ software (version 2.0.45) for QC (including bead removal and high-quality singlet selection). PeacoQC algorithm (v.0.99.30) was applied for preprocessing and data were analysed by FlowSOM (v.2.1.8) clustering in R4.0.<br>- R scripts to analyze the mass cytometry data can be found at https://github.com/saeyslab/CYTOF_covid19_study.<br>- Raw scRNA-seq gene expression matrices generated per sample were merged and analysed with the Seurat package (v3.1.4).

For manuscripts utilizing custom algorithms or software that are central to the research but not yet described in published literature, software must be made available to editors and reviewers. We strongly encourage code deposition in a community repository (e.g. GitHub). See the Nature Research guidelines for submitting code & software for further information.

Data

Policy information about availability of data

All manuscripts must include a data availability statement. This statement should provide the following information, where applicable:

- Accession codes, unique identifiers, or web links for publicly available datasets
- A list of figures that have associated raw data
- A description of any restrictions on data availability

The data supporting the findings regarding cytokine and neutrophil activation biomarker experiments, are available within the paper and its supplementary information files. The mass cytometry raw data files are publicly available on FlowRepository with Repository ID FR-FCM-Z2MW (https://flowrepository.org/experiments/2770). Regarding flow cytometry data, the complete set of FCS files has been deposited on FlowRepository with Repository ID FR-FCM-Z2KP and may be downloaded for further analysis from https://flowrepository.org/experiments/2713. Raw sequencing reads of the scRNA-seq experiments generated for this study have been deposited in the EGA European Genome-Phenome Archive database (EGAS00001005039 for PBMC data; EGAS00001004717 for BAL fluid data). Based on SCope, which is an interactive web server for scRNA-seq data visualisation, a download of the scRNA-seq read count matrices is also available at http://covid19.lambrechtslab.org/. Publicly available data that were used to support this study are available from Gene Expression Omnibus GSE150728.

Field-specific reporting

Please select the one below that is the best fit for your research. If you are not sure, read the appropriate sections before making your selection.

- [x] Life sciences
- [ ] Behavioural & social sciences
- [ ] Ecological, evolutionary & environmental sciences

For a reference copy of the document with all sections, see nature.com/documents/nr-reporting-summary-flat.pdf

Life sciences study design

All studies must disclose on these points even when the disclosure is negative.

Sample size	Sample size calculation was not performed, as all COVID-19 patients admitted to the University Hospitals Leuven were eligible for inclusion in this study (with the exception of below stated exclusion criteria). Inclusion was thus performed on a pragmatic basis, given the research setting (which was the peak of the European COVID-19 pandemic).
Data exclusions	Patients with i) active hematological malignancy; ii) active infectious/inflammatory conditions other than COVID-19; iii) calcineurin-inhibitor treatment, or iv) patients or legal representatives unable or unwilling to give informed consent were excluded.
Replication	Analyses of patient and control samples were performed in batch, reducing experimental variability. Study results obtained through different experimental techniques (eg. flow cytometry, mass cytometry) were in agreement. For each patient analyzed only limited amount of blood was available precluding replication of the same assay twice with the same sample.
Randomization	Allocation to study groups was not randomized. Study cohorts were demographically well balanced (Table 1 and suppl. Table 2). Assessment of impact of demographic covariates for a comparison between critically ill COVID-19 and MAS patients is supplied in suppl. Table 3 (using linear regression analysis, demographic covariates based on Del Valle et al.). Our study was not powered however to adjust for potential confounders using robust (multivariable) regression analysis. This is mentioned in the limitation section of the discussion.
Blinding	Investigators were blinded as to the identities of the samples prior to and during analysis. Sample identities were unblinded after quantification was complete for statistical analysis.

Reporting for specific materials, systems and methods

We require information from authors about some types of materials, experimental systems and methods used in many studies. Here, indicate whether each material, system or method listed is relevant to your study. If you are not sure if a list item applies to your research, read the appropriate section before selecting a response.

Materials & experimental systems

n/a	Involved in the study
	☒ Antibodies
☒	☐ Eukaryotic cell lines
☒	☐ Palaeontology and archaeology
☒	☐ Animals and other organisms
	☒ Human research participants
	☒ Clinical data
☒	☐ Dual use research of concern

Methods

n/a	Involved in the study
☒	☐ ChIP-seq
	☒ Flow cytometry
☒	☐ MRI-based neuroimaging

Antibodies

Antibodies used

- Myeloperoxidase polyclonal antibody (ThermoFisher, PA5-16672, lot number VD2980735); dsDNA-peroxidase antibody (from Roche Cell Death Detection ELISA 11544675001, lot number 42815500; and Roche Cell Death Detection ELISA PLUS 11774425001, lot number 40959200); citrullinated histone H3 (Clone 11D3) ELISA (Cayman Chemicals 501620, lot numbers 0548238 and 0580514). LEGEND MAX(TM) Human Myeloperoxidase ELISA kit (Biolegend, 440007, lot number B296078)

- Maxpar Direct Immune Profiling Assay
Manufacturer: Fluidigm; PN: 201325/S00124; #LOT: P19M0204
Antibody (clone)-Mass PN; LOT
CD45 (HI30)-89Y
CD196/CCR6 (G034E3)-141Pr
CD123 (6H6)-143Nd
CD19 (HIB19)-144Nd
CD4 (RPA-T4)-145Nd
CD8a (RPA-T8)-146Nd
CD11c (Bu15)-147Sm
CD16 (3G8)- 148Nd
CD45RO (UCHL1)-149Sm
CD45RA (HI100)- 150Nd
CD161 (HP-3G10)- 151Eu
CD194/CCR4 (L291H4)-152Sm
CD25 (BC96)-153Eu
CD27 (O323)-154Sm
CD57 (HCD57)-155Gd
CD183/CXCR3 (G025H7)-156Gd
CD185/CXCR5 (J252D4)-158Gd
CD28 (CD28.2)-160Gd
CD38 (HB-7)-161Dy
CD56/NCAM (NCAM16.2)-163Dy
TCRγδ (B1)-164Dy
CD294 (BM16)-166Er
CD197/CCR7 (G043H7)-167Er
CD14 (63D3)-168Er
CD3 (UCHT1)-170Er
CD20 (2H7)-171Yb
CD66b (G10F5)-172Yb
HLA-DR (LN3)-173Yb
IgD (IA6-2)-174Yb
CD127 (A019D5)-176Yb
Cell-ID Intercalator-103Rh

CD11a (HI111)-142Nd 3142006B; 3241804
CD69 (FN50)-162Dy 3162001B; 0832006
CD163 (GHI/61)-165Ho 3165017B; 2631808
NKG2A (Z199)-169Tm 3169013B; 2801902
PD-1 (EH12.2H7)-175Lu 3175008B; 3451808
CD11b (ICRF44)-209Bi 3209003B; 1322021

- FACS:
Fixable viability dye eFluor780; eBioscience, San Diego, CA, USA; fluorochrome-conjugated antibodies against surface markers: anti-CD14 (TuK4), anti-CCR7 (G043H7) (eBioscience); anti-CD3 (REA613) (Miltenyi Biotec, Bergisch Gladbach, Germany); anti-CD4 (SK3), anti-CD8 (SK1), anti-PD1 (EH12.1), anti-CD45RA (HI100) (all from BD Biosciences, San Jose, CA, USA); and anti-CD25 (BC96), anti-HLA-

DR (L243), anti-CD40L (24–31), anti-4-1BB (4B4-1), anti-CD19 (HIB19) (all from BioLegend, San Diego, CA, USA); anti-IFNγ (4S.B3), anti-IL-6 (MQ2-13A5), anti-IL17a (N49-653), anti-RORγt (Q21-559), anti-IL-2 (MQ1-17H12), anti-IL-10 (JES3-9D7), anti-T-bet (4B10), anti-CTLA-4 (BNI3), anti-GATA3 (L50-823) (all from BD Biosciences); anti-IL-4 (MP4-25D2), anti-TNFα (Mab11), anti-FOXP3 (206D) (all from BioLegend).

Validation

- Myeloperoxidase antibody validated in-house with myeloperoxidase standard from Biolegend MPO ELISA (lot number B296078) and with in vitro generated neutrophil extracellular traps. Validated by manufacturer for use in human ELISA applications, including PMA-induced neutrophil extracellular traps.
dsDNA-peroxidase antibody and citrullinated histone H3 ELISA validated in-house with in vitro-generated neutrophil extracellular traps.
Human myeloperoxidase ELISA kit analytically validated by manufacturer for use with human plasma samples for a range from 0.16-10 ng/ml.

- All antibodies for the flow cytometry analyses were obtained from commercial retailers.
They were all validated by the manufacturers and data are available on their website. All antibodies and flow panels were validated in-house by single antibody titration and FMO assays on multiple samples. Further validation of the panel is available in (Neumann J., et al., CTI, 2020, PMID: PMID: 33209300).

- Mass cytometry: antibodies were part of a previously validated commercial kit (Maxpar Direct Immune Profiling Assay (DIPA) kit from Fluidigm©). The assay performance was validated in whole-blood (WB) samples and PBMCs by the manufacturer at 3 different levels (ref. FLDM-400247 Rev 01; https://www.fluidigm.com/binaries/content/assets/fluidigm/white-papers/maxpar-direct-immune-profiling-assay_whitepaper_.pdf?utm_source=Maxpar&utm_medium=White%20Paper&utm_campaign=Maxpar%20Dee%20Immune%20Profiling&utm_content=Deep%20Immune%20Profiling%20with%20the%20Maxpa%20Direct%20Immune%20Profiling%20System). Intra-assay reproducibility was followed by assessments for intermediate precision and inter-site reproducibility. Shortly, eight different assay tubes were used to stain whole-blood samples derived by a single donor. The mean, standard deviation, coefficient of variation, and 95% confidence interval of the mean were calculated for all 35 identified populations. This showed that the %CV of the mean for populations with a frequency ^{3}5% in WB samples is <12%. Three different technicians used three MDIPA tubes to stain WB samples derived by a single donor. The probes were analyzed on 2 or 3 different Helios instruments to quantify the frequencies of the 35 immune populations. This trial was performed to obtain the degree of intermediate precision. For all populations ^{3}5% in frequency in WB samples, the %CV of the mean was <4%. The inter-site reproducibility was tested in three different sites during a two-day period and three other sites during a second two-day period. At each site, a single technician was provided with fresh WB sample by a single donor, which subsequently was stained in triplicate. The data was analyzed at each site's Helios instrument. For populations ≥5% in frequency, the inter-site reproducibility was confirmed by the %CV of the mean <10% in WB specimens. Additional antibodies not included in the Maxpar DIPA kit were titrated on WB and RBC-free samples from donors to determine the optimal staining index.

Human research participants

Policy information about studies involving human research participants

Population characteristics

See Table 1 and suppl. Table 2.

Recruitment

In this prospective single-centre study, adult COVID-19 patients were recruited at the COVID-19 hospitalisation wards of our tertiary care centre in Leuven (Belgium) between March 27th and April 17th 2020. COVID-19 was defined as a positive qRT-PCR on respiratory sample and/or CT imaging compatible with SARS-CoV-2 disease. Patients with i) active hematological malignancy; ii) active infectious/inflammatory conditions other than COVID-19; iii) calcineurin-inhibitor treatment, or iv) patients or legal representatives unable or unwilling to give informed consent were excluded. The control population consisted of i) 10 healthy controls recruited among hospital staff (negative COVID-19 serology); ii) a historical cohort of 10 patients with macrophage activation syndrome (MAS) and iii) 11 patients with non-COVID pneumonia.
As all patients were recruited from our tertiary care centre, this might have introduced a selection bias, which is addressed in the limitations section.

Ethics oversight

All study procedures were approved by the Ethics Committee of the University Hospitals Leuven. Informed consent was obtained from all individuals or their legal guardians.

Note that full information on the approval of the study protocol must also be provided in the manuscript.

Clinical data

Policy information about clinical studies

All manuscripts should comply with the ICMJEguidelines for publication of clinical research and a completedCONSORT checklist must be included with all submissions.

Clinical trial registration

NCT04327570

Study protocol

https://clinicaltrials.gov/ct2/show/NCT04327570

Data collection

Demographic, clinical, laboratory, radiologic, treatment and outcome data from patient electronic medical records (KWS v.3.3.0) were obtained through a standardized research form in Research Electronic Data Capture Software (REDCAP v.10.6.13, Vanderbilt University). All patients were recruited at the COVID-19 hospitalisation wards of our tertiary care centre in Leuven (Belgium) between March 27th and April 17th 2020. Relevant data were collected during the same timeframe, with outcome data evaluation until May 4th.

Outcomes

Outcome measures were not defined. This is a descriptive study, only taking into account clinical characteristics at the time of

sampling.

Flow Cytometry

Plots

Confirm that:

- [x] The axis labels state the marker and fluorochrome used (e.g. CD4-FITC).
- [x] The axis scales are clearly visible. Include numbers along axes only for bottom left plot of group (a 'group' is an analysis of identical markers).
- [x] All plots are contour plots with outliers or pseudocolor plots.
- [x] A numerical value for number of cells or percentage (with statistics) is provided.

Methodology

Sample preparation	Frozen PBMCs were thawed, incubated with phorbol myristate acetate (50 ng/mL), ionomycin (500 ng/mL) and Brefeldin A (8 µg/mL) for 4 hours and subsequently stained after washing steps.
Instrument	BD Symphony.
Software	Manual gating strategy has been performed with FlowJo (version 10.6.1). Compensation was performed using Autospill ref: doi: https://doi.org/10.1101/2020.06.29.177196.
Cell population abundance	Up to 5x105 cells were acquired for each samples.
Gating strategy	Classical manual strategy was applied and cell subsets were defined by well described surface markers.

- [x] Tick this box to confirm that a figure exemplifying the gating strategy is provided in the Supplementary Information.
